# First Insight into the Degradome of *Aspergillus ochraceus*: Novel Secreted Peptidases and Their Inhibitors

**DOI:** 10.3390/ijms25137121

**Published:** 2024-06-28

**Authors:** Anna Shestakova, Artem Fatkulin, Daria Surkova, Alexander Osmolovskiy, Elizaveta Popova

**Affiliations:** 1 Department of Microbiology, Lomonosov MSU, Moscow 119234, Russia; shestakovaaa@my.msu.ru (A.S.); aosmol@mail.ru (A.O.); 2 Laboratory of Molecular Physiology, HSE University, Moscow 101000, Russia

**Keywords:** peptidases, proteases, degradome, MEROPS, HMMER, *Aspergillus ochraceus*, microscopic fungi, pathogenic fungi, antifungal therapy, substrate specificity

## Abstract

*Aspergillus* fungi constitute a pivotal element within ecosystems, serving as both contributors of biologically active compounds and harboring the potential to cause various diseases across living organisms. The organism’s proteolytic enzyme complex, termed the degradome, acts as an intermediary in its dynamic interaction with the surrounding environment. Using techniques such as genome and transcriptome sequencing, alongside protein prediction methodologies, we identified putative extracellular peptidases within *Aspergillus ochraceus* VKM-F4104D. Following manual annotation procedures, a total of 11 aspartic, 2 cysteine, 2 glutamic, 21 serine, 1 threonine, and 21 metallopeptidases were attributed to the extracellular degradome of *A. ochraceus* VKM-F4104D. Among them are enzymes with promising applications in biotechnology, potential targets and agents for antifungal therapy, and microbial antagonism factors. Thus, additional functionalities of the extracellular degradome, extending beyond mere protein substrate digestion for nutritional purposes, were demonstrated.

## 1. Introduction

Microscopic fungi are ubiquitous and extremely important for ecosystems and human organisms [1,2]. They are major destructors of organic matter in soils and valuable producers of different substances for pharmacology and industry. However, mold fungi also contribute to food and feed poisoning and cause human and animal infections [3,4]. For nutrition, substrate attachment, victim invasion, extrolyte (secondary metabolite) biosynthesis, and other processes, these organisms produce proteolytic enzymes [5,6,7,8]. These enzymes have diverse and complex structures and cleave different bonds. In 1996, Rawlings and Barrett introduced the MEROPS database and laid the foundation of peptidase classification based on a multifactorial approach (e.g., homology, origin, catalytic mechanisms, structures) [9]. Later, they highlighted that the term “peptidase” is a correct synonym for “proteolytic enzyme”, because “proteases” come from “cleaving proteins” rather than peptide bonds, and some proteolytic enzymes only cleave peptide bonds in peptides [10]. Thus, in this article, we refer to proteolytic enzymes as peptidases and encourage other scientists to use the correct terminology.

The degradome is defined as the entirety of proteases expressed by an organism. Degradomics encompasses all genomic and proteomic methodologies utilized to identify, characterize, and elucidate peptidases, their substrates, and inhibitors [11]. Extracellular peptidases of microscopic fungi are crucial not only to the nutritional dynamics of soil ecosystems but also to fungal pathogenesis and toxicity, food spoilage, and inter- as well as intraspecific interactions [12]. Generally, a single peptidase of a fungus is described in a single article, representing the major peptidase observed under specific experimental conditions [13]. This approach permits an accurate description of an individual enzyme but falls short of providing comprehensive insight into its biosynthesis and physiological role. Moreover, studies that involve cultivation on varied media types usually do not confirm whether the same or different enzymes were produced under the described conditions [14,15]. To avert the generation of misleading or insufficient data, comprehensive and multifactorial research is necessitated. 

In 2021, Dong et al. conducted a study that mapped the *Aspergillus niger* degradome and identified at least 232 putative peptidases, including those that were intracellular, extracellular, and membrane-localized [16]. This work showed that most of the enzymes encoded by *A. niger* have yet to be described. However, it is not certain that every peptidase found in the genome is expressed, what its exact function is, and whether it contributes significantly to the process it is involved in. In this study, we integrated automatic genomic and transcriptomic approaches with manual checks and literature searches to minimize the rate of errors as far as possible. Further, we endeavored to identify, classify, and characterize the expressed peptidases, elucidating their substrate preferences and potential functions.

A strain of *Aspergillus ochraceus* VKM-F4104D was chosen as a research object, as this species is widely distributed, saprotrophic, and known as a producer of bioactive compounds and extrolites (especially Ochratoxin A) [17,18,19]. Previously, we isolated a peptidase from the VKM-F4104D strain’s culture fluid and showed its unique ability to activate protein C by limited proteolysis [20]. However, some other peptidases of the species have been described [21,22,23], and we decided to illustrate a complete repertoire of the species’ secreted peptidases. A primary hypothesis posited is that among those encoded, only a few major extracellular peptidases are expressed, and these are often isolated and described (alkaline endopeptidases) [24,25]. 

In this work, the genome and transcriptome of *Aspergillus ochraceus* VKM-F4104D are sequenced and every encoded and expressed extracellular peptidase is described after additional manual annotation. Novel potential functions of these peptidases are proposed, providing insights into their physiological roles. A global view of a set of proteolytic enzymes produced by *A. ochraceus* is provided, thereby facilitating a proper understanding of its biology and providing background for further research in toxicology, antifungal therapy, biodegradation, and other fields of science. 

## 2. Results

### 2.1. Aspergillus ochraceus VKM-F4104D Proteolytic Profile

**Figure 1:**
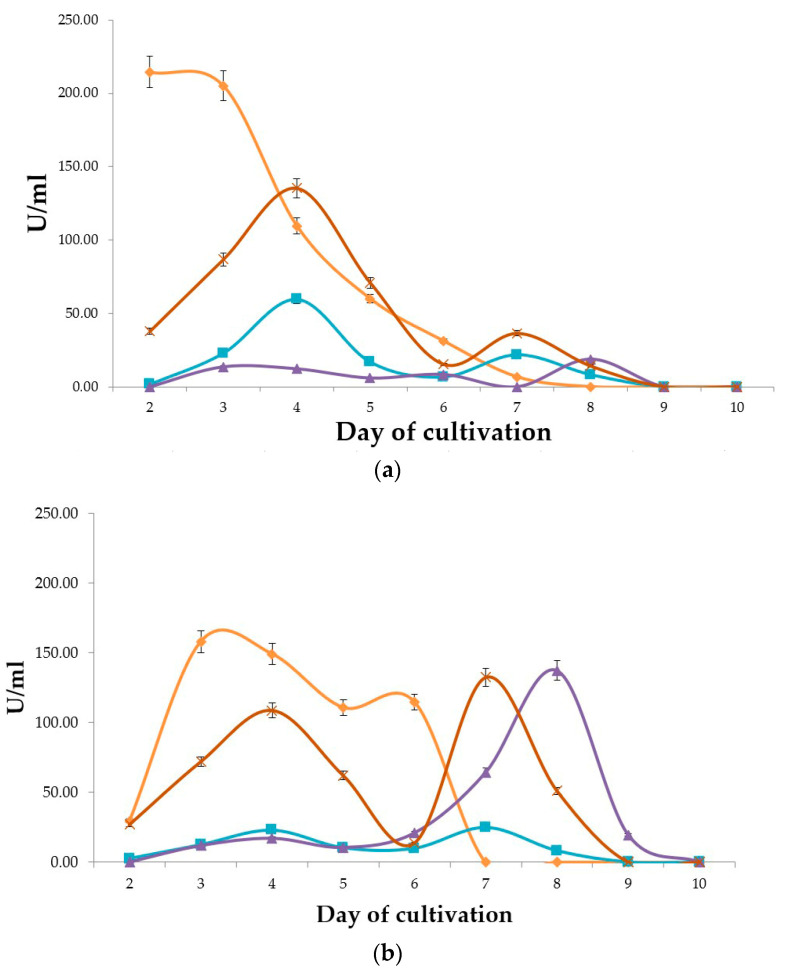
Proteolytic profiles of *A. ochraceus* VKM-F4104D grown with bovine collagen and hydrolyzed fish meal (**a**); bovine collagen and NaNO_3_ (**b**). Legend: 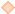, orange—general proteolytic activity; 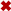, brown—collagenolytic activity; ■, blue—fibrinolytic activity; ▲, violet—elastinolytic activity.

To obtain a proteolytic profile of *A. ochraceus* VKM-F4104D, it was cultivated with two different nitrogen sources (bovine collagen and hydrolyzed fish meal, bovine collagen and NaNO_3_). Every day since the second, general proteolytic, collagenolytic, fibrinolytic, and elastinolytic activities were determined. The proteolytic profiles are illustrated in Figure 1.

Proteolytic profiling confirmed that *A. ochraceus* VKM-F4104D may produce peptidases with activities against different substrates. It was also observed that when combined with collagen, NaNO_3_ does not suppress peptidase biosynthesis; however, it has a significant impact on peptidase production. We were hoping to observe the maximum potential number of peptidases expressed, and therefore, chose a point in time when general proteolytic activity had already begun to decrease and a combination of specific peptidases was potentially the highest (medium N1 on the 4th day of cultivation).

### 2.2. Genome and Transcriptome Sequencing and Mining

To find potentially encoded peptidases of *A. ochraceus* VKM-F4104D, the biomass of the strain cultivated for 4 days in the N1 medium was used for DNA and RNA isolation and further genome and transcriptome sequencing. After sequencing, assembly, and peptidase prediction, every potential peptidase was carefully verified and either assigned to the extracellular part of the degradome or not. In total, 64 extracellular peptidases were predicted for *A. ochraceus* VKM-F4104D. They were classified into six groups based on their catalytic type: aspartic (A), cysteine (C), glutamic (G), metallo- (M), serine (S), and threonine (T). No extracellular asparagine (N), mixed (P), or unknown (U) peptidases were predicted. Cysteine, serine, and metallopeptidases were additionally subclassified into families, thereby providing a more precise analysis of their mechanism of action and potential functions.

Mapping predicted peptidases on the sequenced transcriptome of the fungus surprisingly showed that every predicted peptidase was expressed under the experimental conditions.

#### 2.2.1. Aspartic Peptidases

**Figure 2:**
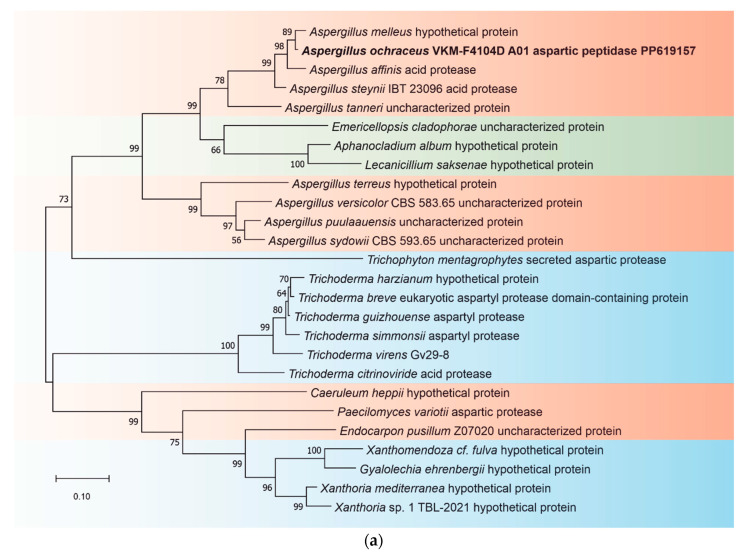

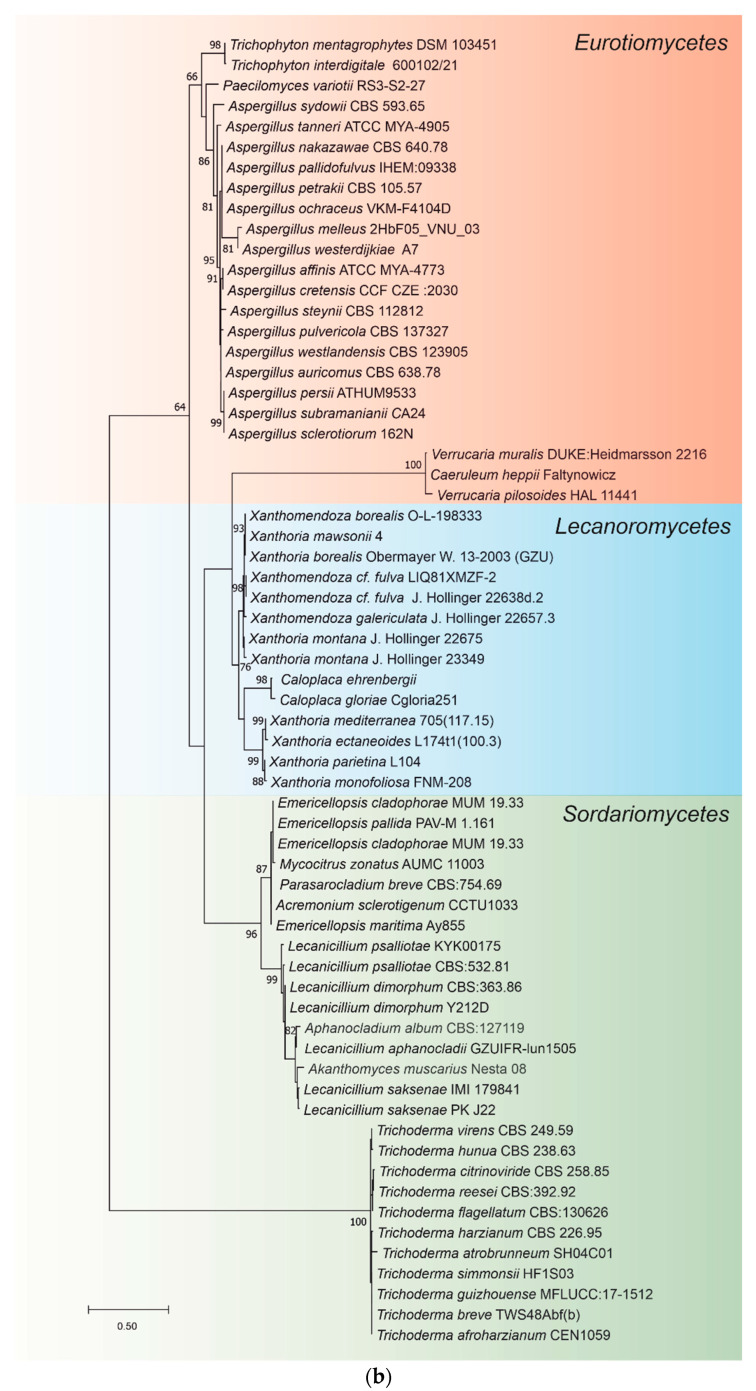
A maximum-likelihood phylogenetic dendrogram based on sequences of A01 peptidase sequence (PP619157) and its homologous sequences (**a**) and ITS regions (**b**). Bootstrapping was performed using 1000 replications. Only values above 60% are indicated.

In total, 11 extracellular aspartic peptidases were predicted for *A. ochraceus* VKM-F4104D, and every enzyme was classified as a member of the A01 (pepsin) family. Two of them, PP619162 and PP619163, were found to be similar (>90%) to previously annotated pepsin-like or acid peptidases of other *Aspergillus* fungi, two others, PP619158 and PP619164, were homologous (>85%) to acid peptidases of aspergilli and somewhat similar (>50%) to a putative pepsinogen of *Biscogniauxia marginata* (KAI1504979.1), a known plant pathogen [26]. Four others (PP619155, PP619157, PP619159, PP619161), despite not being similar to known pepsin-like peptidases, were related to known acid peptidases of different fungi. In addition, PP619157 had homologs (>50%) in other distant fungal taxa, e.g., *Aphanocladium album*, *Emericellopsis cladophorae*, and *Lecanicillium saksenae* (Figure 2).

Three other peptidases did not match any known pepsin-like or acid peptidases.

#### 2.2.2. Cysteine Peptidases

Four different peptidases with cysteine active centers and signals of extracellular localization were found to be encoded in the *A*. *ochraceus* VKM-F4104D genome. InterProScan showed that PP619165 has a GPI-linking domain in addition to a signal peptide for extracellular localization, and belongs to the C13 family of peptidases. Another extracellular peptidase, PP619166, was classified as a member of the C26 family, believed to be a gamma-glutamyl hydrolase. However, in addition to a signal peptide and a glutamine amidotransferase-like domain, InterProScan also predicted an AraC-family transcriptional regulator domain for this enzyme.

Another peptidase, PP619167, belonging to the C40 family, has a predicted cysteine-based peptidoglycan DL-endopeptidase domain. The last predicted extracellular cysteine peptidase of *A. ochraceus* VKM-F4104D, PP619168, was considered part of the C69 family, which comprises dipeptidyl peptidases and aminopeptidases. Interestingly, the found enzyme was homologous to penicillin acylases.

#### 2.2.3. Glutamic Peptidases

In the *A. ochraceus* VKM-F4104D genome, two extracellular glutamic peptidases were found, both belonging to the G01 family. This family comprises fungal glutamic endopeptidases, and sometimes its members are called pepsins 2, pepsins B, or PepB (in contrast to aspartic pepsins), or eqolisins (coming from the E and Q letters for glutamic acid and glutamine in the active site, respectively).

Among those found, the amino acid sequence of one peptidase, PP619169, showed similarity (>94%) to known aspergillopepsins of *A. melleus*, *A. steynii*, and *A. affinis* (XP_052953233.1). Running the sequence of the second enzyme, PP619170, through the InterPro database also resulted in finding a concanavalin A-like lectin/glucanase domain.

#### 2.2.4. Metallopeptidases

Initially, a total of 25 metallopeptidases were predicted for A. ochraceus VKM-F4104D. Three peptidases (PP619176, PP619177, PP619178) were classified as members of the M06, M105, and M106 families, respectively. Two peptidases were expected to belong to the M12 family: PP619179 was found to be homologous to the Zn-dependent (zincin) metallopeptidase of *Fusarium longipes* (RGP81324.1), and PP619180—to the zincin of *Aspergillus steynii* (XP_024701532). PP619181 was classified as a member of the M14 family and showed significant similarity (>90%) to the pseudopeptidase Ecm14 of *Aspergillus melleus* (KAK1145959.1). Three peptidases (PP619182, PP619183, PP619184) were proposed to be a part of the M20 family and had dimerization and ArgE-DapE-like domains. Family M28 was represented by five peptidases. Among those, PP619188 and PP619189 were classified as aminopeptidases, while for the other three (PP619185, PP619186, PP619187), the targeted end of the action was not identified. However, as members of the M28 family, they are exopeptidases. Interestingly, PP619185 also had a glutaminyl-peptide cyclotransferase domain. The most represented family of metallopeptidases of *A. ochraceus* VKM-F4104D was M35, with eight members (PP619190, PP619191, PP619192, PP619193, PP619194, PP619195, PP619196, PP619197). Enzymes of this family are also called deuterolysins. For each predicted deuterolysin, a collagenase domain was also described. In addition to deuterolysin, one fungalysin (a member of the family M36, PP619198) was predicted. The last presented family was M72, with two members, both of them had an ADAM (“a disintegrin and metalloprotease”) domain.

Thus, automated prediction resulted in finding 25 putative extracellular metallopeptidases in the genome of *A. ochraceus* VKM-F4104D.

#### 2.2.5. Serine Peptidases

The initial prediction resulted in revealing 71 potential serine extracellular peptidases belonging to the families S8, S9, S10, S15, S28, S33, and S41, suggesting serine peptidases to be the most represented. However, different domain organizations and non-proteolytic functions were predicted for many enzymes, e.g., an alpha-1,2-mannosidase domain for PP619216, a polyketide transferase domain for PP619222, and others. To identify non-peptidase homologs (such as esterases, deacetylases, non-catalytic proteins, etc.), an additional study was conducted using the InterPro service. This made it possible to exclude 49 predicted hydrolases, as they are characterized by changes either directly in the active center or in its environment, excluding the possibility of proteolytic activity. Each of them had an α/β-hydrolase domain, likely affecting the prediction result. It should be noted that most non-peptidase homologs have been shown to hydrolyze esters.

#### 2.2.6. Threonine Peptidases

Only one extracellular threonine peptidase was found to be encoded in the genome of *A. ochraceus* VKM-F4104D. A BLAST search revealed that many species of *Aspergillus* and *Penicillium* fungi have homologous (70%>) proteins annotated as gamma-glutamyl transpeptidase (e.g., of *A. minisclerotigenes*) and nucleophile aminohydrolase (e.g., of *A. novoparasiticus*).

#### 2.2.7. Peptidase Inhibitors

The MEROPS database includes not only peptidases but also their inhibitors of different families. For *A. ochraceus* VKM-F4104D, five inhibitors belonging to different families were predicted. Among them, four proteins were similar to structurally annotated ones of *A. melleus*, *A. affinis*, and *A. steynii* (N8T08_007383, KD926_010906, and P170DRAFT_356083, respectively). However, no functional annotation was available for these proteins, thus making it impossible to predict their real functions or confirm inhibitory activity. These proteins were classified as I02 (PP619171), I07 (PP619172, PP619173), and I44 (PP619175) families of inhibitors. The fifth protein, PP619174, was homologous to a known peptidase inhibitor from *Penicillium griseofulvum* (XP_040647989.1), and InterProScan predicted the presence of an S8-inhibiting domain for this protein.

## 3. Discussion

### 3.1. Principles of Degradomics

The degradome is a complete set of peptidases within an organism; yet, it is the extracellular peptidases that assume principal significance in nutritional processes and environmental interplay. Consequently, our investigation solely scrutinized the extracellular repertoire of the *A. ochraceus* VKM-F4104D degradome. Simultaneously, to ensure a more rational evaluation, membrane-bound enzymes were deliberately excluded, while embracing enzymes exhibiting multifaceted enzymatic activities beyond mere proteolysis. For instance, encompassing broad-spectrum serine hydrolases, usually referred to as “serine peptidases”, alongside amidolytic enzymes capable of degrading substrates other than proteins was imperative, as they contribute to the holistic proteolytic profile of the fungus.

When predicting the peptidases, we intentionally set the cut-off to include more proteins satisfying the model, as this approach decreases the probability of losing potential enzymes, despite requiring more effort in manually checking the structural organization of the predictions. This strategy was shown to be rewarding, as some of the proteins that we further classified as peptidases were homologous to proteins with unknown functions; others were described as “hydrolases”, and we managed to narrow and specify these descriptions.

Additionally, we described five potential peptidase inhibitors, sometimes also referred to as part of the degradome [11]. Usually, only exogenous peptidase inhibitors of fungi are studied, and this may provide insight not only into fungal physiology but also into the development of new antifungal drugs [27,28].

Proteolytic profiling of *A. ochraceus* VKM-F4104D has shown that peptidase biosynthesis depends on the composition of the nutritional media; therefore, the functions and specializations of the produced enzymes are diverse and probably depend on the majority and nature of substrates to be cleaved. Supporting transcriptome sequencing surprisingly indicated that every extracellular peptidase encoded in the genome was expressed at its own rate.

A typical extracellular peptidase has three domains: a signal peptide (usually at the N-terminus), a propeptide, and a catalytic domain [29]. The propeptide prevents a peptidase from premature activation and is cleaved during maturation. A signal peptide with propeptide is cleaved off by Kex2-like peptidases (kexins or proprotein convertases) in the Golgi apparatus, and the mature enzyme is subsequently released into the extracellular space [30,31]. Interestingly, kexins belong to the S08 family and have a distinguishable specificity for arginine and lysine in the P1 and P2 positions, respectively. These enzymes may self-cleave if the peptidases change their conformation, e.g., due to a pH change (aspartic peptidases), or other mature peptidases’ actions. Peptidases also have a highly conserved active center, and disruptions or mutations in the active center cause a loss of the enzyme’s function. This pattern was shown for pseudopeptidases (proteins structurally similar to peptidases but lacking catalytic function). Additionally, MEROPS contains not only peptidases but also their inactive or non-proteolytic homologs. Therefore, our manual annotation included verification of potential catalytic centers with those typical for the corresponding family.

Another complication in the degradome characterization involved the annotation of serine peptidases. Most of the predicted enzymes belong to the SC clan (S9, S10, S15, S28, S33) and are α/β-hydrolases [32]. Members of this group are distinguished by a unique fold: the core of each enzyme is folded into eight β-sheets connected by α-helices [33]. These proteins evolved from a common ancestor group, but they developed substrate specializations: some hydrolases may act as dehalogenases, haloperoxidases, esterases, peptidases, and even lyases [34]. For serine peptidases, however, retaining esterase activity in addition to proteolytic is known [35]. This strategy is efficient when a combination of proteinaceous and esteric substrates is available in the environment, as it requires fewer biosynthetic processes and less regulation, and explains why peptidases still harbor additional activity in contrast to other members of this clan. These complex evolutionary and structural relationships were the reason to check not only domain organization but also the catalytic center of our predicted enzymes. Despite being more time consuming, this approach enabled proper characterization and annotation of the α/β-hydrolases of *A. ochraceus* VKM-F4104D.

### 3.2. Aspartic Peptidases

Pepsin-like (A01 family) peptidases are believed to be synthesized as propeptides, thereby providing additional protection from unwanted self-proteolysis [36]. Fungal pepsin-like peptidases are also known to be produced as zymogens. For aspartic peptidases, a change in pH is responsible for the autocatalytic transformation to active peptidases [31]. Aspartic peptidases are thought to have a low pH optimum and are sometimes automatically annotated as “acidic proteases” (according to our BLAST results of predicted enzymes). Thus, their contribution to a species’ degradome is especially significant when the environmental pH is acidic. In general, aspartic peptidases with neutral pH optima are also known [37]. However, during the preparation of this article, no works describing microbial neutral aspartic peptidases were found. An additional check with EpHod has shown that for *A. ochraceus*, every predicted A01 peptidase has an estimated pH optimum lower than 4.5 (Table 1). Thus, the automatic annotation of extracellular fungal aspartic peptidases as “acid peptidases” is reasonable.

Interestingly, when analyzing MEROPS data with SignalP, we found that the number of A01 family peptidases for *A. ochraceus* is equal to that known for *A. niger* (11) and different from those of *A. clavatus* (6) and *A. flavus* (13); hence, showing that the number of aspergillopepsins may differ depending on the taxonomy of the species (the listed species belong to sections *Circumdati*, *Nigri*, *Clavati*, and *Flavi*, respectively).

Additionally, many aspartic peptidases form dimers during maturation [38]. This is a typical feature of A02 family members, but A01 peptidases may also dimerize (e.g., pepsin itself) [39]. For aspergillopepsins Cho et al. demonstrated that dimerization occurs with the help of hydrogen bonds [40]. However, at low pH, the concentration of protons increases, leading to the protonation of ionizable groups such as the carboxyl groups of aspartic acid and glutamic acid, as well as the amino groups of lysine and arginine. This protonation can disrupt hydrogen bonding interactions by introducing positive charges, which can repel each other, or by altering the electrostatic environment of the hydrogen bond, weakening its strength. However, while preparing this article, we did not find any aspartic peptidase isolated and purified in its dimer form. Probably, the bonds stabilizing the dimer are weak, or conventional protein purification methods are too harsh.

Another interesting observation is that peptidase PP619157 is homologous to aspartic peptidases of fungi from other classes (Eurotiomycetes and Sordariomycetes); however, it is not found in closely related fungi. Rawlings previously showed that some microbial peptidases may be found in unusual species, mainly due to horizontal gene transfer (HGT) [41]. Hence, simultaneously finding homologous sequences in relatively close and distant species suggests two explanations: either this peptidase was transferred via HGT, or it originated from an ancestor and was lost in some descendant taxa.

### 3.3. Cysteine Peptidases

Some biochemical characteristics of the cysteine peptidases from *A. ochraceus* VKM-F4104D are listed in the Table 2.

When running PP619165 through InterProScan, we found a signal of extracellular localization, as well as a region indicating that the protein is embedded in the membrane. Functionally, it was predicted to be a GPI-anchor transamidase. GPI-anchor transamidases are located in the endoplasmic reticulum (ER) and are involved in transferring a GPI anchor to a protein by transamidation [42]. As a general principle of protein biosynthesis, a membrane-embedded protein is incorporated in the lipid bilayer during its translation. Thus, we assume that PP619165 is indeed an ER-located enzyme, and therefore, exclude it from the extracellular part of the degradome.

We also additionally checked PP619166, which had glutamine amidotransferase (GATase) and an AraC transcription factor regulator domains. AraC-mediated regulation does not involve proteolysis or transamidation [43], and it is supposed to act intranuclearly. However, the encoded PP619166 has a signal of extracellular localization, rather than transport to the nucleus. We suggest that this enzyme may have occurred as a consequence of mutation, and its real function is yet to be determined. Therefore, we excluded it from the degradome of *A. ochraceus*, but agree that future research aimed at clarifying its real function may alter this decision.

Another peptidase found, PP619167, belonging to the C40 family, also had a peptidoglycan DL-endopeptidase domain. Enzymes with similar hydrolytic activity are typically of bacterial origin and are necessary for cell wall remodeling [44,45]. We have not found any fungal peptidase for which a similar activity was described, probably because substrates imitating bacterial cell wall components are not common. We suggest that this peptidase is involved in microbial antagonism, and it helps to deliver fungal antibacterial antibiotics into the victim cell. Peptidases, and fungal peptidases in particular, are found to be useful agents for bacterial biofilm disruption [46,47]. Accordingly, the found peptidase of *A. ochraceus* VKM-F4104D might help in solving the biofilm formation issue, and our future research will be aimed at expressing and purifying this enzyme for further testing in biofilm destruction.

The fourth predicted extracellular cysteine peptidase of *A. ochraceus* VKM-F4104D was a member of the C69 family and classified as a dipeptidyl peptidase or aminopeptidase. This enzyme, like other extracellular peptidases with similar catalytic features, is probably involved in later stages of protein degradation, and its action results in dipeptide and single-amino acid release. Interestingly, bacterial peptidases of the same family and catalytic type may form octamers [48]. This likely increases the efficiency of peptidolytis but is harder to produce and regulate. Generally, extracellular microbial enzymes are postulated to be small and monomeric, and every exception is unique and should be examined.

### 3.4. Glutamic Peptidases

Compared to aspartic peptidases, glutamic peptidases have similar catalytic mechanisms and often have comparable (acidic) pH optima [49,50]. We found that *A. ochraceus* VKM-F4104D expresses two glutamic peptidases (Table 3).

These peptidases are known for their unique yet simple fold, and in 2018 it was shown that occurrence of functional redundancy and diversification of glutamic peptidases resulted in multiple originations of their paralogs [51]. The same research suggested that eqolisins may have originated from prokaryotic organisms by HGT. We found the nucleotide sequences of both peptidases in the genome and discovered that there were no introns in these sequences, which supports the hypothesis of their HGT origin. This hypothesis additionally explains why glutamic peptidases are less frequent than aspartic ones, as they arose later.

Yet, the regulation of aspartic and glutamic peptidase biosynthesis is complicated: at least one aspergillopepsin (of the first or the second type, aspartic or glutamic, respectively) is required for fungal growth at low pH [52]. It is unclear whether these enzymes compete with or complement each other. Our additional check indicated that the pH optima for glutamic peptidases of *A. ochraceus* VKM-F4104D are 5.7 for PP619169 and 6.2 for PP619170, which is higher than the corresponding characteristics of aspartic ones. We may suggest that at acidic pH, aspartic peptidases play a major role in substrate degradation, and later, when the environmental pH is increasing due to desamination, glutamic peptidases are expressed, as their pH optima are less acidic than those of aspartic peptidases.

For PP619170, InterProScan predicted a concanavalin A-like lectin/glucanase domain. The function of this domain depends on the specific protein context in which it is found. However, this domain typically possesses two distinct functional activities: lectin activity and glucanase activity. Lectins are carbohydrate-binding proteins that recognize and bind to specific sugar moieties on glycoproteins or glycolipids. The concanavalin A-like lectin domain can bind to carbohydrates. This lectin activity is often involved in various biological processes, including cell–cell adhesion, signaling, immune response modulation, and pathogen recognition; and, in addition to its lectin activity, the concanavalin A-like domain may also possess glucanase activity [53]. Glucanases are enzymes that catalyze the hydrolysis of glycosidic bonds in glucans, which are polysaccharides composed of glucose monomers. Glucanases play important roles in various biological processes, including plant cell wall degradation, microbial cell wall remodeling, and carbohydrate metabolism [54,55]. It was demonstrated for *A. fumigatus*, that glucanases are involved in cell lysis, branching, and cross-linking [56]. Recently, Zhou et al. confirmed that concanavalin A-like lectin/glucanase of *Penicillium expansum* (Peclg) is necessary for growth, stress response, and the virulence of this strain [57]. Thus, it probably plays the same role in other microscopic fungi, including *Aspergillus*. A combination of these activities may be essential for the pathogenesis and invasion of species with proteins and glucans in their cell walls. However, such a combination of a concanavalin A-like lectin/glucanase domain with a proteolytic domain is still unique, as this “multifunctional enzyme” is extracellular.

### 3.5. Metallopeptidases

Metallopeptidases are peptidases typically incorporating a metal ion coordinated by two histidines and a glutamate. Most of the metallopeptidases are monozinc enzymes (so-called ‘zincins’); however, the zinc may naturally be substituted with Co (II) or Mn (II) [58]. According to MEROPS, metallopeptidases are subdivided into 106 families, and for *A. ochraceus* VKM-F4104D, we initially predicted 25 extracellular metallopeptidases belonging to 10 of them (M06, M12, M14, M20, M28, M35, M36, M72, M105, M106). Among those found, all peptidases are likely to have a Zn (II) ion in their active center, rather than any other metal. Being the second most represented group of peptidases after serine ones, they have a variety of different functions in the fungus. However, many of them are not detected by standard methods due to their substrate specificity, or are not synthesized or folded at all, as many laboratory nutrition media are prepared using ultrapure water and no zinc salts are added [13]. Interestingly, zinc in the environment may stimulate the biosynthesis of peptidases of different catalytic types, not increasing the proportion of metallopeptidases in the degradome [59].

PP619176 was classified as a member of the M06 (endopeptidase) family. In mammal-, insect-, and nematode-invading bacteria, M06 peptidases act as virulence factors [60]. They cleave host proteins during infection, mostly operating as immune inhibitors [61]. Fungal M06 peptidases are not properly studied; however, Pfavayi et al. have shown that an M06 peptidase of *A. fumigatus* is an allergen for patients sensitized to fungi [62]. Therefore, we suggest that in fungi, the extracellular M06 peptidases also contribute to pathogen–host interaction, and thus, may be a probable target for antifungal therapy.

The M12 family of metallopeptidases contains zinc-dependent endopeptidases. The best-studied enzymes of this family are snake venom peptidases (reprolysins) [63]. They cause the formation of thrombi by activating coagulation factors [64]. In addition, snake venom peptidases have high collagenolytic activity [65]. *A. ochraceus* VKM-F4104D produces two extracellular metallopeptidases of the M12 family. Both of them, PP619201 and PP619180, have a predicted collagenase catalytic domain, and thus, are putative collagenases. For bacterial M12 peptidase, collagenolytic (and even elastinolytic) activity was demonstrated [66]. Usually, fungal collagenolytic and elastinolytic peptidases are either not assigned to families during studies [67], or belong to the M35 and M36 families [68]. However, as collagenolysis and elastinolysis are initial steps of host–pathogen interaction, studying these putative collagenases is important.

Additional checks on PP619181 (initially predicted as a member of M14) have shown that this protein does not have a catalytic domain. Moreover, it is highly similar (98%) to Ecm14 of *A. melleus*. Ecm14 is a pseudopeptidase conservative throughout the Ascomycota phylum. McDonald et al. investigated the function of Ecm14 in *Saccharomyces cerevisiae* and suggested that it is involved in cell wall maintenance and/or fungal invasion [69]. As they noted, mammalian M14 pseudopeptidases bind collagen and other matrix proteins [70,71]. Therefore, Ecm14 might be involved in substrate binding or pathogenicity. As this protein has no catalytic domain or potential active site and is similar to a known pseudopeptidase, we are not counting it as a part of the *A.ochraceus* VKM-F4104D degradome.

The M20 family contains Zn-dependent exopeptidases with different patterns of action: carboxypeptidases, dipeptilpeptidases, and aminopeptidases. These enzymes have a very flexible catalytic domain: in addition to peptidase activity, they may perform hydrolysis of other substances. Some peptidases may be active against rare amino acid combinations; for example, the M20 peptidase from *A. oryzae* (CdpA) can cleave cysteinyl-glycine bonds (Cys-Gly) [72]. For each M20 peptidase in this work (PP619182, PP619183, PP619184), the presence of an ArgE-DapE-like domain was found. ArgE cleaves a variety of N-acyl-amino acid substrates, whereas the DapE domain is considered to be a meso-diaminopimelic acid (mDAP)-producing enzyme [73]. For activation, ArgE-DapE requires dimerization. Indeed, for all three peptidases, a dimerization domain was also found [74]. In bacteria and some fungi, DapE is also involved in lysine and mDAP biosynthesis, where succinic acid is a precursor. Ascomycetes and Basidiomycetes possess another pathway of lysine biosynthesis, involving l-α-aminoadipate [75]. However, in “higher” fungi, e.g., *Valsa mali* or *Colletotrichum graminicola*, ArgE-DapE-containing proteins are also found. Yin et al. have shown that they arose via HGT. They also illustrated that *V. mali* has six out of nine encoded proteins involved in the succinylase pathway, and suggested that the incomplete DAP pathway may still work and thereby supply fungi with intermediates produced by surrounding microorganisms. This strategy provides a competitive benefit to collecting nitrogen substances from the environment [76]. *A. ochraceus* has three enzymes with the desired action, thus, it probably gives it an advantage over other species in the community it inhabits.

The family M28 is considered unusual, as it includes both carboxy- and aminopeptidases. Recently, a zinc-dependent cobalt-stimulated M28 peptidase was found and described for bacteria [77]; however, fungal peptidases of the M28 family have not been properly studied. It was shown that M28 aminopeptidase expression depends on the cultivation conditions for *Trichoderma harzianum* CECT 2413 [78], thereby suggesting its important and diverse functional role. Among M28 peptidases of *A. ochraceus*, PP619189 and PP619188 were manually annotated as aminopeptidases (the former had a fungal aminopeptidase domain, and the latter has a 99% homology to aminopeptidase of *A. tanneri* (XP_033421378.1)). For PP619187 and PP619186, only their belonging to the exopeptidase type was shown and further manual analysis did not result in a more precise classification. Furthermore, PP619185 was identified as a glutaminyl-peptide cyclotransferase (QPCT), believed to convert glutamic acid to pyroglutamic acid at the N-terminal of the molecule. The latter is more stable and resistant to proteolysis, and its presence increases the stability of non-cytoplasmic proteins [79]. Fungal QPCTs have not previously been described, and as they contribute to enhancing protein stability outside the cell, they may enforce fungal virulence. Thus, if the targets of QPCTs are proteins involved in invasion and/or pathogen–host interaction, their inhibitors are potential antifungal drugs, and further characterization of fungal QPCTs’ is essential.

M35, or the deuterolysin family, contains peptidases with high affinity to basic proteins, including protamines and histones. However, conventional laboratory substrates are not suitable for determining deuterolysin activity, and therefore, they are often overlooked and not revealed in traditional enzymatic studies [80]. Collagen of different types is believed to have a rather high isoelectric point, varying from 7.2 to 8.5 [81,82], and thus, is positively charged at acidic and neutral pH. Elastin is another component of connective tissue and it is highly resistant to proteolysis due to intramolecular non-peptide bonds formed by lysine oxidases [83]. Elastin and collagen constitute a significant part of lung tissues. It is believed that elastases are the main virulence factors of lung-invading fungi; however, we suggest that collagenases may have the same role. The oxygen-rich lamellar bodies, which usually confront fungi during the infection process, are believed to have moderately acidic pH [84,85]. Recently, Chen et al. showed that for pathogenic *Trichophyton* spp., expression of deuterolysins increases at low pH, which is beneficial for deuterolysin action [86]. For *Coccidioides* spp., another pathogenic genus, the importance of M35 peptidases in host invasion was also discovered previously [87]. Considering the aforementioned, we should emphasize the importance of the M35 family peptidases in studying fungal pathogenicity and virulence. The collagenolytic activity exhibited by fungi warrants detail-oriented consideration in fungal studies due to its potential pathogenic implications. The abundance of deuterolysin gene copies might correlate with the invasive nature of fungal species or strains. Moreover, deeper insights into the evolutionary trajectory of this gene family hold promise for elucidating the intricacies underlying pathogen–host interactions.

In addition to deuterolysins, crucial for overcoming the tissue barriers of the host, one fungalysin from the family M36 was found in the genome of *A. ochraceus* VKM-F4104D. M36 is a family of peptidases predominantly found in pathogenic fungi and capable of degrading matrix proteins, e.g., laminin and keratin (both essential components of tissues) [88]. Laminin plays a significant role in respiratory tract development and is expressed in the lungs throughout life [89], while keratin fills dead keratinocytes, comprising the major mass of the outer layer of the epidermis [90]. Possibly, deuterolysins and fungalysins act together regardless of the invasion path (respiratory or skin), and fungalysins initiate this process by disrupting the outer layers of skin while deuterolysins act in later stages, where collagen and elastin are predominant. Recent studies confirm that the number of genes and activity of fungalysin is increased for pathogenic fungi, including dermatophytes [91,92]. Peptidase PP619198 is the only fungalysin of *A. ochraceus*, and this fact decreases the probability of its potential pathogenicity, which corresponds to the results of biosafety tests previously performed for this isolate [93].

The M72 family, according to the MEROPS database, contains extracellular metallopeptidases. However, InterProScan predicted an ADAM domain and a transmembrane domain at the C-terminus of the protein for both M72 peptidases of A. ochraceus [94]. Since ADAM peptidases are truly membrane-integrated, we decided to exclude both of the found enzymes, PP619199 and PP619200, from the extracellular degradome. However, they definitely contribute to substrate degradation. Fungal mycelia, penetrating and wrapping substrate particles, may benefit from membrane-incorporated enzymes, as they facilitate direct substrate–harvester interaction. Pathogenic fungi may additionally use it for victim intrusion [5] through the process called “ectodomain shedding”, where peptidases cleave membrane-bound proteins at positions near the cell surface [95]. Considering that many molecules involved in cell signaling and immune response are integrated into the membrane, ADAM peptidases are probably essential for escaping from the immune system of the victim.

Families M105 and M106 have a significant number of non-peptidase homologs, and therefore, we carefully checked their structure and organization. Both PP619177 (M105) and PP619178 (M106) contain an endopeptidase catalytic domain and are predicted to be collagenases. However, a zinc-coordinating (active) center in PP619177 is distorted, and instead of H230, E231, H234, and H240, it only has H230, while other positions are filled with amino acid residues not even biochemically close to the original ones. Thus, it is a non-peptidase homolog, and we excluded it from the degradome of *A. ochraceus*. In contrast, the active site of PP619178 was complete, but the M106 family is not properly described, and its amino acid sequence had a functionally uncharacterized DUF6055 domain found in bacteria and fungi, and no additional information on these functions is available and further investigations are necessary.

Thus, *A. ochraceus* VKM-F4104D has 21 extracellular metallopeptidases belonging to seven families (M06, M12, M20, M28, M35, M36, M106). Some of their biochemical features are provided in the Table 4.

### 3.6. Serine Peptidases

Serine peptidases are the most represented peptidases in living organisms [96]. Initially, we predicted 71 extracellular serine peptidases in three different clans: SB (family S8), SC (families S9, S10, S15, S28, S33), and SK (family S41). However, further analysis significantly changed our primary results, as most of the enzymes appeared to lack peptidase activity. Authentic serine peptidases of *A. ochraceus* VKM-F4104D and their biochemical features are provided in the Table 5.

Enzymes of the SC clan are believed to have evolved from a common ancestor and have a distinctive feature of an α/β-hydrolase fold. They were described in 1992 as enzymes that do not show significant homology in functions but have a similar domain and structural organization, consisting of eight β-sheets connected by α-helices [33]. During our HMM-based prediction, we uncovered 66 potential serine peptidases with α/β-hydrolase fold. However, only 16 of them had a non-distorted active center of the peptidase type and others were excluded from the degradome of *A. ochraceus* VKM-F4104D. This high rate of false positive results may have arisen due to the HMMER application for peptidase prediction, as it is exactly based on structural organization rather than homology. Mistaken for peptidases, many enzymes with an α/β-hydrolase fold had active centers and domain organization similar to other members of this clan. For example, we found potential feruloyl esterases (PP619217, PP619231, PP619262), tannases (PP619232, PP619254), polyketide transferases (PP619228, PP619222), a cutinase (PP619263), and other esterases or non-defined α/β-hydrolases. Among the enzymes of the SC clan that we counted as extracellular peptidases, there were members of the S10, S28, and S33 families.

It is noteworthy, that among 36 putative S9 peptidases, only 1 (PP619234) had a peptidase domain. However, it also had a DPP6 (dipeptidyl peptidase-like protein 6) domain, and serine in its active center was substituted by glutamate [97]. These enzymes are known to be α/β-hydrolases with a disrupted active center, and their function is not clear. Therefore, we regard PP619234 as a pseudopeptidase and exclude it from the degradome of *A. ochraceus* VKM-F4104D.

Among the predicted members of the S10 family, each enzyme indeed had a peptidase domain and active center. Seven of them were serine carboxypeptidases (PP619241, PP619246, PP619247, PP619244, PP619243, PP619240, PP619248). These enzymes, sometimes referred to as “Y carboxypeptidases”, as they were first described for yeasts, have exceptional serine peptidases with acidic pH optima [98]. For the listed enzymes, the predicted pH optima does not exceed 5. These enzymes are also unique in their ability to release proline, thereby significantly contributing to nutrition. This was proved by Muszewska et al., who showed that for symbiotic fungal species, the number of S10 peptidases is significantly reduced [99]. In dermatophytes, such as *Trichophyton rubrum*, S10 peptidases are GPI-linked and may have keratinolytic activity, playing the role of an invasion factor [100]. Thus, the number and localization of S10 peptidases are dictated by the lifestyle of a fungus.

Another member of this family, PP619245, has a “low-temperature requirement A” (LtrA) domain. This domain is found in proteins essential for bacterial growth at low temperatures [101] and in some fungal proteins. However, its function in fungi is still not clarified.

The least found S10 peptidase, PP619242, in addition to the active site of the peptidase and a signal of extracellular localization, had WD-40 (or beta-transducin repeats), and a G-protein beta WD-40 repeat, in particular. This domain and subdomain are involved in a variety of functions, such as signal transmission, transcription and cell cycle regulation, apoptosis, mating, and others [102]. However, we cannot suggest any reasonable explanation of its function, and further research is certainly required for clarification of this.

Two proteins, PP619250 and PP619249, were initially assigned to the S15 family. However, we did not observe a typical S15 catalytic triad for either of them, and therefore, excluded them from the degradome of *A. ochraceus* VKM-F4104D. Interestingly, PP619249 has a carboxyl-esterase domain, which proves complex evolution relationships and urgent accuracy in predicting and annotation of α/β-hydrolases.

Peptidase family S28, found only in eukaryotes, contains exopeptidases that selectively hydrolyze prolyl bonds, releasing dipeptides from the C-terminus of the protein. For *A. ochraceus* VKM-F4104D, we predicted three active S28 peptidases. Each of them, PP619252, PP619253, and PP619251, has a catalytic domain and was unambiguously assigned to the extracellular degradome of the fungus. It was shown that S28 members assist in protein degradation, but are not major. Using *A. fumigatus* as a model, Sriranganadane et al. have shown that S28 peptidases are secreted in both acidic and alkaline conditions, and release X-X-Pro and X-X-X-Pro peptides, thereby facilitating protein degradation by other peptidases incapable of cleaving prolyl bonds [103]. Another study additionally confirmed that S28 peptidases are involved in the degradation of large and prolyl-rich proteins [104], thereby suggesting their importance for collagen degradation, as it is rich in proline residues. This fact may be either used for studying fungal invasion, or for industrial application (e.g., in leather processing or cosmeceutics). The last family of the SC clan is the S33 peptidase family. Initially, we predicted 16 extracellular peptidases, but only three of them (PP619266, PP619258, and PP619260) had peptidase homologs and peptidase domains. Similarly to the S28 family, S33 family members may release single amino acid residues and small peptides, cleaving prolyl bonds, but from the N-terminus, rather than the C-terminus of the protein. Interestingly, their system functions are probably slightly different: S28 and S33 peptidases are both less distributed among pathogenic fungi, but in plant-associated fungi, prolyl aminopeptidases (S33) are abundant [99]. S33 peptidases are considered the most expressed overall and sometimes are suggested as markers of saprotrophy [105], and their contribution to global substrate degradation is definitely underestimated.

Another important family of peptidases found in *A. ochraceus* VKM-F4104D is family S8. This family, also known as the subtilisin family, consists of endopeptidases with Asp, His, and Ser residues in the active center. It is divided into S8A (subtilisin-like) and S8B (kexin-like) subfamilies. In one of our previous works [106], we found, described, and cloned an alkaline peptidase of *A. ochraceus* VKM-F4104D (peptidase activator of protein C of human blood plasma). A closer look at its structure confirmed that it belongs to the S8A subfamily of serine peptidases, and is indeed an endopeptidase. We previously showed [107] that it is synthesized in response to the presence of proteins in the environment, and thus, we suppose that the major function of this enzyme is related to nutrition and primary substrate proteolysis. *A. ochraceus* VKM-F4104D has three extracellular S8 peptidases: PP619201 and PP619203 (S8A, subtilisin-like) and PP619202 (S8B, kexin-like).

Extracellular subtilisins are frequently reported to be characterized for *Aspergillus* fungi, and for many of them, their roles in nutrition and pathogenesis have already been shown [108,109,110,111,112]. They are less specific and easily detectable with conventionally used casein and azocasein, which may explain their abundance and easier characterization compared to peptidases of other families. Moreover, they might be the most widespread in the degradome, as they are known as primary antigens for humans [113,114]. Thus, understanding the biosynthesis and functional peculiarities of S8A peptidases is crucially important, not only for physiology research but also for fungal allergology and immune response studies. Interestingly, fungal S8A peptidases are usually reported as alkaline peptidases, and both PP619201 and PP619203 have a pH optimum of 9.1–9.5.

S8B peptidases, in contrast to S8A, are believed to have regulatory rather than nutritional functions. Kexins catalyze zymogen processing and thereby facilitate protein maturation, cleaving bonds following a pair of dibasic residues [96]. However, regulatory functions are typically intracellular, and finding an extracellular kexin was unexpected. In 2016, it was reported that disruption of extracellular kexin caused cell wall distortion in *A. oryzae* [115]. This suggests that extracellular S8B peptidases are involved in the regulation of different secretory pathways, including propeptide-based checkpoints [116].

Peptidase clan SK contains three families of serine peptidases (S14, S41, S49). Among them, *A. ochraceus* VKM-F4104D has three extracellular peptidases of the family S41 (also known as the C-terminal processing peptidase family). PP619272, PP619270, and PP619271 belong to S41A subfamily. These peptidases recognize the C-terminus of the protein, but cleave bonds distant from the recognition site. Intracellularly, these peptidases are responsible for the degradation of improperly folded proteins and are involved in autolysis and cell death. Likely, extracellular S41 peptidases only possess the latter function. It was shown that this family is ancient and conserved in Ascomycota, and is involved in fungal–plant interactions, where it acts as an avirulence factor, causing an immune response in plants possessing the resistance gene [117]. However, further deeper analysis and research involving gene knockout is required to verify its function in *Aspergillus* and *A. ochraceus* VKM-F4104D, in particular.

### 3.7. Threonine Peptidases

One extracellular threonine peptidase encoded in the genome of *A. ochraceus* VKM-F4104D was classified as a member of the T03 family (Table 6) and is believed to be a self-processing peptidase, incorporating both aminopeptidase and aminotransferase activities, which is unusual, as these enzymes are conventionally classified in two separate groups (EC 3.4 for peptidases and EC 2.3 for acyltransferases) [118]. In addition, other peptidases of the T03 family may cleave, in addition to peptide bonds, a variety of other bonds, e.g., amide bond in N-linked protein glycosylation or in sphingolipid complexes [119,120,121]. Thus, *A. ochraceus* VKM-F4104D extracellular peptidase from the T03 family either acts as an extracellular transglutaminase, or amino(amido-)peptidase or has both activities. However, we suggest counting it as a part of the degradome, as it contributes to protein degradation.

Furthermore, the role of this protein may be more complicated than it seems. Recently, Mamun and Maryama first reported the function of fungal intracellular transglutaminase—this enzyme is involved in wound-related protection of the organism, creating cross-links between proteins at the septal pore [122]. However, the function of extracellular transglutaminase is still unclear, as septa are formed intracellularly [123]. Another interesting observation is that regardless of the type of reaction catalyzed by T03 family peptidases, ammonia is released [124]. Thus, T03 peptidases contribute to desamination and increase the pH of the extracellular environment. Additionally, both glutamine and glutamate may be taken up from the outside of the fungal cell [125]; however, only glutamine is known as a regulator of nitrogen catabolism (along with ammonia and asparagine [126], and its presence represses proteins involved in inorganic nitrogen uptake [127]. It was reported that an organic nitrogen source is more preferable for fungi rather than inorganic [128]. However, we cannot explain the relationships between T03 peptidase and its role in nitrogen catabolism regulation.

### 3.8. Peptidase Inhibitors

Peptidase inhibitors are synthesized by microscopic organisms along with peptidases to prevent potential autolysis. As there are more than 100 extracellular peptidases, we may suggest that synthesized inhibitors either have a broad specificity or act towards the most active or valuable peptidase. However, since the structures of the predicted enzymes are highly diverse, the former is less probable. A found PP619174 was annotated as an S8-peptidase inhibitor. The S8 family mostly contains serine subtilisin-like endopeptidases with alkaline pH optima. Therefore, it probably represents the most contributive part of a fungal degradome. It is known that premature subtilisin peptidases act as inhibitors and chaperons for themselves [129,130]; however, the peptide only contained 104 amino acid residues (without a signal peptide), and neither the homologous proteins found among the predicted S08 peptidases of *A. ochraceus* VKM-F4104D, nor InterProScan or BLASTp showed any protein with a similar domain. Thus, this peptidase inhibitor probably only conserved inhibitory activity, but may have originated from a subtilisin peptidase. To further clarify its origin and functions, evolutionary genomics and recombinant protein approaches may be used.

Other predicted inhibitory peptides cannot be unambiguously assigned to the degradome of *A. ochraceus* VKM-F4104D, as the manual checks of their structural annotation did not result in finding described proteins with such a function. However, PP619175, predicted as a member of the I44 family, according to MEROPS, is supposed to be an inhibitor of metallocarboxypeptidases in family M14. And, as described, *A. ochraceus* VKM-F4104D possesses one (PP619181), therefore, they may be complementary to each other. Other predicted inhibitors were assigned to I02 and I07 families, which are mainly described as serine trypsin-peptidase inhibitors. However, no trypsin-like extracellular peptidases were found for the studied species. In any case, further studies are required to determine the real function and activity of predicted fungal peptidase inhibitors. These putative inhibitors or their homologs were not previously described, and therefore, they may represent a novel class of antifungal drugs, as peptidases are known to be major invasion factors in aspergillosis cases [5,131,132].

### 3.9. Degradome of A. ochraceus VKM-F4104D

Some characteristics of the degradomes of various *Aspergillus* species, encapsulating the findings derived from this work, are presented in Table 7.

Despite all the predicted peptidases being expressed, we cannot claim that they are translated, matured, and active at the exact time of analysis. Using transcriptomic and proteomic techniques, Budak et al. showed that 17 to 41% of the encoded peptidases are present and may be detected in the culture liquid [133]. This proves that peptidases act in complexes and complement each other’s action, and are precisely regulated. It is known that peptidase biosynthesis depends on different factors, such as carbon and nitrogen sources, temperature, pH of the medium, aeration, and others [6,134]. For further clarification of the entangled interactions and roles of each enzyme in fungal life, additional experiments involving combined differential transcriptomics and LS-MS approaches are required in each particular case [135]. In addition, extracellular enzyme production depends not only on the translation of the protein itself, but also on other factors, e.g., proteins involved in vesicle transport or post-translational modifications, and complex study of the cell as a complex micro-factory of peptidases is the most appropriate approach.

**Figure 3:**
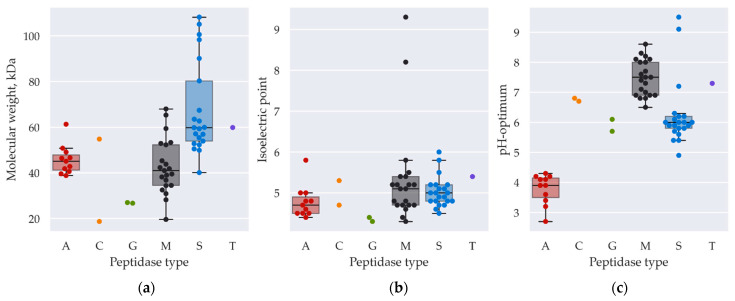
Box plots illustrating molecular weights (**a**), isoelectric points (**b**), and optimal pH (**c**) of mature predicted peptidases of *A. ochraceus* VKM-F4104D. A—aspartic, C—cysteine, G—glutamate, M—metallo-, S—serine, T—threonine peptidases.

Some biochemical features of the found peptidases may also promote the unraveling of their functions and physiological roles. Using the amino acid sequences of the peptidases (without signal sequences), we calculated their molecular weights, isoelectric points, and pH optima (Figure 3).

Thus, the molecular mass of peptidases differs from 18 to 150 kDa, with a median of around 50.2 kDa. Such a molecular weight promotes easier expression and folding, which is beneficial for the cell. PP619167 has the lowest molecular mass among those predicted (18 kDa) and is a bacterial-cell-wall-modifying enzyme. Such a small size may facilitate flexibility in substrate adhesion and docking, promoting better hydrolysis. Another family of small proteins is the M35 family (deuterolysin family). Their masses are between 19.6 and 52.3 kDa with a mean of 36.0 kDa, and such a size may help in overcoming barriers of tertiary protein structures. Among the largest peptidases of *A. ochraceus* VKM-F4104D, serine carboxypeptidases of the S10 and S41 families are prevalent.

The isoelectric points of most peptidases are lower than 6, and this may be useful for protein purification from culture fluid when using chromatography. Two enzymes with alkaline isoelectric points are zinzin PP619179 and deuterolysin PP619194.

The predicted pH optima for peptidases of *A. ochraceus* VKM-F4104D are generally clustered by peptidase catalytic type. As supposed, aspartic peptidases are most active at low pH, while serine and metallopeptidases are alkaline. Interestingly, the pH optima of the glutamic peptidases are higher than those of the aspartic and lower than those of the serine and metallopeptidases, and thus, these peptidases may be intensively produced when the pH of the medium is slightly acidic. Cysteine peptidases’ pH optima, as supposed, are neutral. Only one extracellular threonine peptidase, PP619274, has a neutral pH optimum, which corresponds to its potential function of medium alkalinization by desamination.

Thus, peptidases are clustered according to their biochemical properties, which correlate with their physiological roles and allow us to conclude the fitness and adaptations of the fungus to the environment.

## 4. Materials and Methods

### 4.1. Submerged Fermentation of Aspergillus ochraceus VKM-F4104D

For submerged fermentation of the strain, a two-stage technique was used. Seven-day-old cultures grown on slant malt extract agar (MEA) were used to prepare a spore suspension (10^5^ spores/mL), and subsequently, inoculated into the pre-culture medium (g/L: malt extract (Carl Roth, Muhlburg, Germany)—67.0, glucose (Sigma-Aldrich, Burlington, VT, USA)—20.0, peptone (Merck, Burlington, VT, USA)—1.0). After 48 h of cultivation, 3 mL of the biomass was transferred to fermentation media [136]. It is known that microbial hydrolases may be either inducible or constitutive [137]. To induce as many peptidases as possible, two media differing in the nitrogen source were used. Both media contained the same basic composition (g/L: glucose (Sigma-Aldrich, Burlington, VT, USA)—30.0, glycerol (Merck, Burlington, VT, USA)—70.0, NaCl (Sigma-Aldrich, Burlington, VT, USA)—0.5, KH_2_PO_4_ (Sigma-Aldrich, Burlington, VT, USA)– 0.5, MgSO_4_ (Sigma-Aldrich, Burlington, VT, USA)—0.5), and the N1 medium additionally contained hydrolyzed fish meal (DiaM, Moscow, Russia) and bovine collagen (PanEco, Moscow, Russia) (2.5 and 4.0 g/L, respectively), while the N2 medium incorporated bovine collagen (PanEco, Moscow, Russia) and NaNO_3_ (Sigma-Aldrich, Burlington, VT, USA) (4.0 and 2.0 g/L, respectively). This choice of media composition was dictated by conventional principles of fungal nutrition: hydrolyzed proteins are easier to degrade, and thus, may induce oligo-, carboxy-, and aminopeptidases; collagen is a fibrous protein of diverse microstructure, and for its proteolysis, diverse endopeptidases are required; NaNO_3_ also acts as a nitrogen source and may be used to observe non-inducible (constitutive) peptidases [5].

For fermentation (repeated in triplicate), 100 mL of the selected medium was poured into 750 mL Erlenmeyer flasks, and cultures were agitated at 200 rpm and 28 ± 0.2 °C in shaker-incubator (Biosan, Riga, Latvia). Every day, from the second day, samples were collected, filtered with a glass filter to separate mycelia, and used for determination of proteolytic activity. After day 4, the biomass obtained from the flasks of N1 medium was mixed and used for subsequent DNA and RNA isolation.

### 4.2. Proteolytic Activity Determination

Four conventional substrates—azocasein (Sigma-Aldrich, Burlington, VT, USA), azocoll (Merck, Burlington, VT, USA), fibrin (Sigma-Aldrich, Burlington, VT, USA), and a chromogenic peptide substrate of elastase S-4760 (N-Succinyl-Ala-Ala-Ala-p-nitroanilide) (Sigma-Aldrich, Burlington, VT, USA) —were prepared on 100 mM Tris-HCl buffer (pH 7.0). To initiate the reaction, 200 μL of 0.2% azocasein or azocoll, or 1.0% fibrin was mixed with 100 μL of sample. The azocaseinolytic reaction was stopped after 30 min by adding 300 μL of 10% trichloroacetic acid (TCA) (DiaM, Moscow, Russia). The azocollagenolytic reaction was stopped after 30 min by adding 300 μL of 0.4% (0.1N) NaOH (Sigma-Aldrich, Burlington, VT, USA). The fibrinolytic reaction was stopped after 60 min by adding 300 μL of 10% TCA. The tubes were subsequently centrifuged at maximum speed (14,300 rpm) on an Eppendorf MiniSpin centrifuge (Eppendorf, Hamburg, Germany) to precipitate the sediment. In supernatant, absorbance at 275, 340, or 519 nm was measured for fibrin, azocasein, and azocoll, respectively [138,139]. A unit of fibrinolytic activity was defined as the amount of enzyme releasing 1 μg of tyrosine in 1 min under the reaction conditions. The calibration curve was prepared with tyrosine. A unit of azocollagenolytic and azocaseinolytic activity was determined as the amount (μg) of the respective degraded substrate in 1 min in 1 mL of the reaction mixture. The calibration curve was prepared with pronase and proteinase K.

S-4760 (N-Succinyl-Ala-Ala-Ala-p-nitroanilide) is a chromogenic substrate used for elastinolytic activity assessment [140]. When the substrate is subjected to proteolysis, p-nitroaniline (pNa) is released in the reaction mixture, and its absorbance is later measured spectrophotometrically at 405 nm. To set up the reaction, 150 μL of 0.5% S-4760 solution was mixed with 200 μL of the sample and after 5 min, the reaction was stopped by adding 200 μL of 50% acetic acid (DiaM, Moscow, Russia). The absorbance was subsequently measured spectrophotometrically with a wavelength of 405 nm. One unit of activity was defined as the amount (μM) of released pNa in 1 mL of the reaction mixture in 1 min under the reaction conditions.

### 4.3. DNA and RNA Extraction and Sequencing

For whole-genome sequencing of *A. ochraceus* VKM-F4104D, total DNA was isolated using an MP Biomedicals™ Gnome™ DNA Isolation Kit (MP Biomedicals, Santa-Ana, USA); libraries were prepared with Roche KAPA kit (Roche, Basel, Switzerland), and subsequently run on an Illumina NestSeq sequencer (Illumina, San-Diego, USA). The quality control of the raw readings was performed using FastQC [141]. Genomic and transcriptomic reads were assembled into contigs using SPAdes 3.15.5 [142,143]. Sequencing was performed in BioSpark, Russia. Transcriptomic sequences were additionally preprocessed using fastp [144] and mapped to the *A. ochraceus* VKM-F4104D genome using STAR [145]. The expression level was assessed using RSEM [146]. The assembled genome and transcriptome were deposited at the NCBI databases and received accession numbers JBAFXH000000000 and SRX23611215 (in the SRA), respectively.

### 4.4. Sequence Processing and Protein Prediction and Description

MEROPS data were downloaded and used to construct HMMs for peptidase families [29]. Families with less than 2 sequences were ignored. If the family contained more than 1000 sequences, then the sample size was limited to a random choice of 1000 samples. Subsequently, the sequences were aligned using Clustal Omega (https://www.ebi.ac.uk/jdispatcher/msa/clustalo, accessed on 29 March 2024) [147], and then, transformed into profiles with HMMER [148]. AUGUSTUS 3.5.0 was used to predict genes in the genome of *A. ochraceus* VKM-F4104D. The sequence of predicted proteins was scanned using HMMER 3.4 against a constructed database to detect potential peptidases. SignalP 6.0 [149] was used to identify secreted peptidases. Isoelectric points and molecular weights were estimated using BioPython (https://biopython.org/, accessed on 29 March 2024). pH optima for selected proteins were estimated using an EpHod tool designed by Gado et al. [150]. Subsequently, every predicted protein was manually checked using the Basic Local Alignment Search Tool (BLAST) [151] and InterProScan 98.0 [152]. To show evolutionary interactions, a phylogenetic dendrogram based on sequences of A01 peptidase PP619157 and its homologs was built, along with one based on the ITS regions of the same species. The phylogenetic tree was constructed by the maximum-likelihood method with the MEGA 10.0 software [153]. Bootstrap analysis was performed using 1000 replicates.

## 5. Conclusions

Thus, the extracellular degradome of *A. ochraceus* VKM-F4104D consists of 58 peptidases. Among them, 32 are endopeptidases and 26 are exopeptidases. They were classified according to the MEROPS database as aspartic (11), cysteine (2), glutamic (2), serine (21), threonine (1), and metallopeptidases (21). These peptidases have different functions and participate in nutrition, pathogen–host interaction and invasion, pH regulation, microbial antagonism, cell death and autolysis, and regulation of cell physiology. Thus, understanding fungi’s degradomics, especially those tightly interacting with humans, is key to many biotechnological, pharmacological, and industrial solutions.

## Figures and Tables

**Table 1:** Classification and some biochemical characteristics of the aspartic peptidases from *A. ochraceus* VKM-F4104D.

GenBank ID	Family	Exo-(X) or Endo-(N) Peptidase	TPM ^1^	FPKM ^2^	Molecular Weight	pI	pH Optimum	Length ^3^
PP619154	A01	N	124.72	79.34	61.3	4.7	4.2	564
PP619155	A01	N	116.29	73.97	49.1	4.5	3.4	465
PP619156	A01	N	111.29	70.8	46.6	5.0	3.6	437
PP619157	A01	N	109.93	69.93	45.1	5.8	4.1	399
PP619158	A01	N	107.59	68.44	39.6	4.4	4.2	363
PP619159	A01	N	105.73	67.26	46.4	4.8	3.9	433
PP619160	A01	N	93.13	59.24	41.8	4.8	3.9	380
PP619161	A01	N	69.47	44.19	50.8	4.5	4.3	482
PP619162	A01	N	66.83	42.51	42.8	5.0	3.2	403
PP619163	A01	N	49.16	31.27	38.8	4.6	2.7	368
PP619164	A01	N	45.06	28.66	40.6	4.5	4.1	376

Here and further: ^1^ Transcripts per kilobase million; ^2^ fragments per kilobase million; ^3^ amino acid residues, without signal sequence.

**Table 2:** Classification and some biochemical characteristics of the cysteine peptidases from *A. ochraceus* VKM-F4104D.

GenBank ID	Family	Exo-(X) or Endo-(N) Peptidase	TPM	FPKM	Molecular Weight	pI	pH Optimum	Length
PP619167	C40	X	34.05	21.66	18.7	4.7	6.8	172
PP619168	C69	X	59.29	37.71	54.8	5.3	6.7	492

**Table 3:** Classification and some biochemical characteristics of the glutamic peptidases from *A. ochraceus* VKM-F4104D.

GenBank ID	Family	Exo-(X) or Endo-(N) Peptidase	TPM	FPKM	Molecular Weight	Isoelectric point	pH Optimum	Length
PP619169	G01	N	108.58	69.07	26.7	4.4	5.7	252
PP619170	G01	N	90.78	57.75	27.0	4.3	6.1	254

**Table 4:** Classification and some biochemical characteristics of the metallopeptidases from *A. ochraceus* VKM-F4104D.

GenBank ID	Family	Exo-(X) or Endo-(N) Peptidase	TPM	FPKM	Molecular Weight	pI	pH Optimum	Length
PP619176	M06	N	55.52	35.32	28.2	4.4	7.6	249
PP619179	M12	N	147.88	94.07	30.9	9.3	8.6	279
PP619180	M12	N	82.46	52.46	68.0	5.8	7.5	634
PP619182	M20	X	140.64	89.47	45.3	5.1	6.9	426
PP619183	M20	X	71.20	45.29	42.3	4.7	7.0	402
PP619184	M20	X	68.29	43.44	59.4	5.2	6.8	538
PP619185	M28	X	106.57	67.79	41.8	5.1	6.5	373
PP619186	M28	X	79.98	50.88	41.0	5.5	8.1	368
PP619187	M28	X	70.11	44.60	39.0	5.4	8.0	356
PP619188	M28	X	69.75	44.37	52.9	5.4	6.8	490
PP619189	M28	X	61.40	39.06	53.3	4.7	6.9	490
PP619190	M35	N	151.00	96.06	19.6	4.7	8.3	183
PP619191	M35	N	74.94	47.67	52.3	4.7	8.1	500
PP619192	M35	N	66.27	42.16	34.5	4.8	7.4	327
PP619193	M35	N	56.85	36.17	36.8	4.8	7.7	343
PP619194	M35	N	56.21	35.76	38.2	8.2	8.0	335
PP619195	M35	N	55.48	35.29	32.5	5.2	7.1	285
PP619196	M35	N	52.11	33.15	39.5	5.2	8.2	348
PP619197	M35	N	31.01	19.72	34.4	4.6	7.5	308
PP619198	M36	N	63.84	40.61	65.3	5.2	7.3	604
PP619178	M106	N	97.39	61.95	43.8	4.3	6.9	405

**Table 5:** Classification and some biochemical characteristics of the serine peptidases from *A. ochraceus* VKM-F4104D.

GenBank ID	Family	Exo-(X) or Endo-(N) Peptidase	TPM	FPKM	Molecular Weight	Isoelectric Point	pH Optimum	Length
PP619201	S08	N	91.25	58.04	50.5	5.2	9.1	474
PP619202	S08	N	85.27	54.25	90.1	5.0	7.2	822
PP619203	S08	N	47.50	30.22	40.1	5.8	9.5	384
PP619241	S10	X	104.62	66.55	59.9	4.9	5.9	533
PP619246	S10	X	59.15	37.63	57.1	4.5	5.6	508
PP619247	S10	X	46.38	29.51	63.5	5.2	5.9	580
PP619245	S10	X	59.40	37.79	100.6	4.7	5.8	906
PP619244	S10	X	64.56	41.07	49.9	4.8	5.8	447
PP619243	S10	X	78.54	49.96	52.3	4.7	5.7	472
PP619242	S10	X	79.35	50.48	105.1	5.1	6.0	948
PP619240	S10	X	115.98	73.78	67.4	4.6	5.4	606
PP619248	S10	X	41.65	26.50	59.3	4.9	4.9	535
PP619252	S28	X	104.92	66.74	59.8	4.8	6.0	536
PP619253	S28	X	87.17	55.45	62.7	4.8	5.4	562
PP619251	S28	X	248.74	158.23	98.3	6.0	6.1	873
PP619266	S33	X	52.39	33.33	53.9	5.0	6.2	496
PP619260	S33	X	90.35	57.48	56.9	5.5	6.3	525
PP619258	S33	X	93.88	59.72	52.8	4.8	6.0	477
PP619272	S41	N	37.45	23.82	55.4	5.2	6.2	503
PP619270	S41	N	143.61	91.36	108.2	5.0	6.0	976
PP619271	S41	N	80.87	51.44	80.3	5.1	5.9	730

**Table 6:** Classification and some biochemical characteristics of the threonine peptidase from *A. ochraceus* VKM-F4104D.

GenBank ID	Family	Exo-(X) or Endo-(N) Peptidase	TPM	FPKM	Molecular Weight	Isoelectric Point	pH Optimum	Length
PP619274	T03	X	70.26	44.70	59.9	5.4	7.3	557

**Table 7:** Summary of current putative extracellular peptidases in Aspergilli (based on [133]).

Species	Total Genes	Putative Peptidases	Serine Peptidases	Aspartic Peptidases	Metallo- Peptidases	Other Peptidases
*A. fumigatus* Af293	9781	45	16	9	8	12
*A. flavus* NRLL 3357	12,604	63	23	15	9	16
*A.oryzae* RIB40	12,030	57	21	14	11	11
*A.terreus* NIH 2624	10,406	44	16	9	6	13
*A.fischeri* CBS 544.65	10,406	45	15	11	7	12
*A.nidulans* FGSC A4	10,680	40	13	8	6	13
*A. niger* ATCC 1015	11,162	53	22	15	7	9
*A. ochraceus* VKM-F4104D	11,038	58	21	11	21	5

## Data Availability

The original data presented in the study are openly available in Genbank with accession numbers JBAFXH000000000 (genomic data), SRX23611215 (transcriptomic data) and peptidases’ mRNAs sequences are available under numbers PP619154–PP619274.
